# The efficacy and safety of topical administration of tranexamic acid in spine surgery: a meta-analysis

**DOI:** 10.1186/s13018-018-0815-0

**Published:** 2018-04-24

**Authors:** Wei Luo, Ru-xin Sun, Han Jiang, Xin-long Ma

**Affiliations:** 1 Department of Orthopedics, Tianjin Hospital, Tianjin, 3002111 People’s Republic of China; 2 Department of Gynaecology and Obstetrics, Tianjin Hongqiao Hospital, Tianjin, 300131 People’s Republic of China; 3 Department of Orthopedics, Tianjin Third Central Hospital, Tianjin, 300170 People’s Republic of China

**Keywords:** Tranexamic acid, Spine, Blood loss, Transfusion, Meta-analysis

## Abstract

**Background:**

We conducted a meta-analysis from randomized controlled trials (RCTs) and non-RCTs to assess the efficacy and safety of tranexamic acid (TXA) in spine surgery.

**Methods:**

Potentially relevant academic articles were identified from the Cochrane Library, MEDLINE (1966–2017.11), PubMed (1966–2017.11), Embase (1980–2017.11), and ScienceDirect (1985–2017.11). Secondary sources were identified from the references of the included literature. The pooled data were analyzed using RevMan 5.1.

**Results:**

Three RCTs and one non-RCT met the inclusion criteria. There were significant differences in total blood loss (MD = − 267.53, 95% CI − 373.04 to − 106.02, *P* < 0.00001), drainage volume (MD = − 157.00, 95% CI − 191.17 to − 122.84, *P* < 0.00001), postoperative hemoglobin level (MD = 0.95, 95% CI 0.44 to 1.47, *P* = 0.0003), and length of hospital stay (MD = − 1.42, 95% CI − 1.92 to − 0.93, *P* < 0.00001). No significant differences were found regarding transfusion requirement, deep vein thrombosis (DVT), pulmonary embolism (PE), wound hematoma, and infection between the two groups.

**Conclusions:**

The present meta-analysis indicated that the topical application of TXA in spinal surgery decreases the total blood loss and drainage volume and preserves higher postoperative hemoglobin level without increasing the risk of DVT infection, hematoma, DVT, and PE.

## Background

Spinal surgery is associated with significant perioperative blood loss that may lead to acute anemia and lead to serious complications [1]. Blood transfusions are often required to correct acute anemia and carry their own risks, such as inducing disease transmission, hemolysis, and anaphylactic reactions [2, 3]. In addition, blood transfusions increase the economic burden. Multiple blood conserving interventions [4] have been utilized to minimize the blood loss, such as normovolemic hemodilution, blood salvage, hypotensive anesthesia, bipolar electrocautery, and hemostatic agents. However, many patients still require blood transfusions.

Tranexamic acid (TXA), an antifibrinolytic drug, competitively blocks the lysine-binding site of plasminogen and has been used to reduce blood loss in spine surgery for many years [5]. Various studies have reported that intravenous application of TXA reduces blood loss and allogenic blood transfusions in spinal surgery without increasing related complications [6–8]. In theory, there remains an unresolved concern about the potential thrombogenicity of intravenous TXA [9]. Topical application of TXA would decrease systemic absorption and result in lower risk of adverse reactions. Recently, several studies [10–12] have reported topical application of TXA in spinal surgery. However, there is still no consensus whether to use topical application of TXA during spinal surgery or not. Moreover, some limitations exist in previous studies such as small sample size and inconclusive results. Therefore, we conducted a large sample meta-analysis to evaluate the efficacy and safety of topical application of TXA in spinal surgery from randomized controlled trials (RCTs) and non-RCTs.

## Methods

### Search strategy

**Fig. 1:**
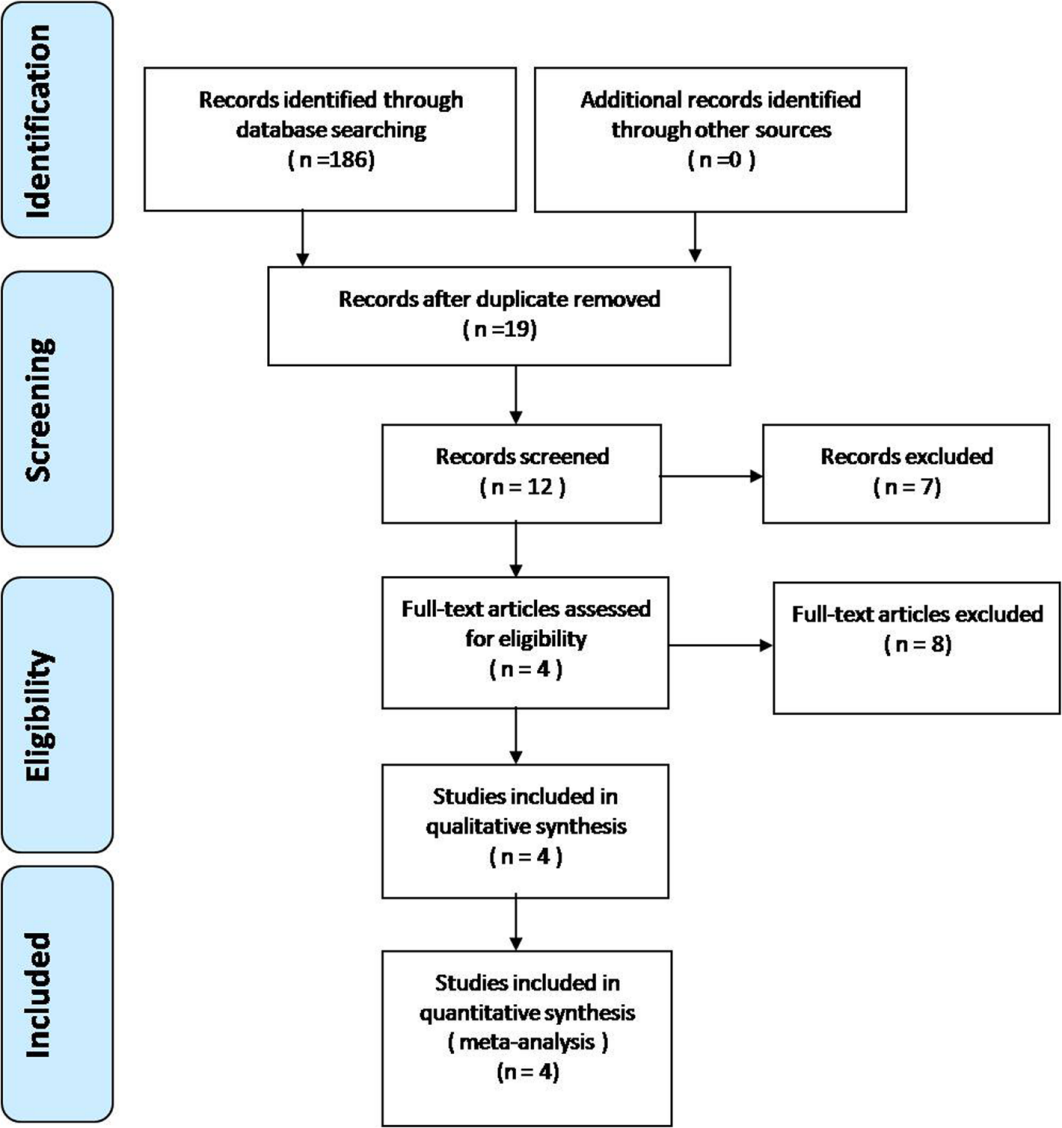
Flowchart of the study selection process

Electronic databases were searched, including Cochrane Library, MEDLINE (1966–2017.11), PubMed (1966–2017.11), Embase (1980–2017.11), and ScienceDirect (1985–2017.11). In addition, the same search terms were manually searched for the reference lists of all included studies, relevant books, review articles, and meeting proceedings to identify trials that might have been missed in the electronic search. The search process was conducted as follows in Fig. 1. The key words “tranexamic acid,” “topical,” “spine,” and “surgery” were used in combination with the Boolean operators AND or OR.

### Inclusion criteria

Studies were considered eligible for inclusion if they met the following criteria: (1) patients treated with spine surgery; (2) the intervention used TXA and studies contained a control group; (3) the outcomes included blood loss, blood transfusion, post-operative Hb level, length of hospital stay, peri-operative outcomes, and complications; and (4) the study was a published or unpublished comparative trial (RCTs or non-RCTs).

### Exclusive criteria

We excluded articles that were (1) studies without controlled groups, (2) articles without available full-text versions, and (3) no available outcomes data.

### Selection criteria

Two reviewers independently screened the titles and abstracts for eligibility criteria. Subsequently, the full text of the studies that potentially met the inclusion criteria were read, and the literature was reviewed to determine the final inclusion. Disagreement was resolved by consulting a third reviewer.

### Quality assessment

According to whether the study is a randomized or non-randomized trial, the methodological index for non-randomized studies (MINORS) form was used to assess retrospective controlled trials [13]. Quality assessment for RCT was conducted according to a modification of the generic evaluation tool used by the Cochrane Bone, Joint and Muscle Trauma Group [14]. Disagreements were resolved by consensus or consultation with the senior reviewer.

### Data extraction

Two researchers independently extracted the data from the included literature. The corresponding author was consulted for details in the case of incomplete data. The following information was extracted: first author name, year of publication, intervening measures, comparable baseline, sample size, and outcome measures. Other relevant parameters were also extracted from individual studies.

### Data analysis and statistical methods

Pooling of data was analyzed by RevMan 5.1 (The Cochrane Collaboration, Oxford, UK). Heterogeneity was estimated depending on the value of *P* and *I*^2^ using the standard chi-square test. When *I*^2^ > 50%, *P* < 0.1 was considered to be a significant heterogeneity. Therefore, a random-effects model was applied for data analysis. A fixed-effects model was used when no significant heterogeneity was found. Subgroup analysis was performed to investigate sources in the case of significant heterogeneity. Mean difference (MD) and 95% confidence interval (CI) were presented for continuous outcomes. Risk difference (RD) and 95% CIs were calculated for dichotomous data.

**Table 1:** Characteristics of included studies

Study	Operation	Cases (T/C)	Mean age (T/C)	Gender (F)	Dosage	Transfusion trigger
Krohn 2002	Lumbar surgery	16/14	51/54	9/9	0.5 g	8 g/dL
Liang 2016	PLIF	30/30	51.13/53.83	15/12	2 g	7 g/dL
Ren 2017	PLIF	50/50	55.2/58.7	20/19	1 g	7 g/dL
Xu 2017	PLIF	40/40	53.1/57.4	21/27	1 g	8 g/dL

*T* tranexamic acid, *C* control, *F* female, PLIF *p*osterior lumbar interbody fusion

## Results

### Search results

A total of 186 studies were identified as potentially relevant literature reports. By scanning title and abstract, 182 reports were excluded according to the eligibility criteria. No additional studies were obtained after the reference review. Ultimately, three RCT [11, 15, 16] and one non-RCT [12] were eligible for data extraction and meta-analysis. The search process is shown in Fig. 1.

### Study characteristics

Demographic characteristics and details concerning the literature type of the included studies are summarized in Table 1. Statistically similar baseline characteristics were observed between both groups.

### Risk of bias assessment

**Fig. 2:**
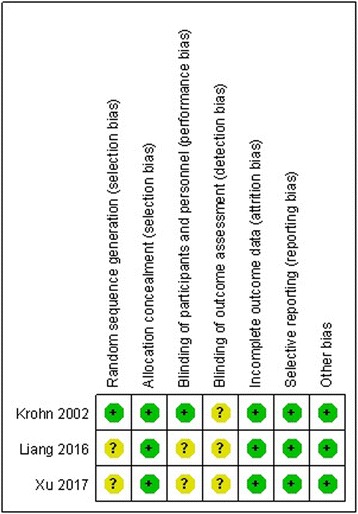
Risk of bias summary of randomized controlled trials

RCT quality was assessed based on the Cochrane Handbook for Systematic Review of Interventions (Fig. 2). For the non-RCTs, the MINORS score is 20. The methodological quality assessment is illustrated in Table 2.

**Table 2:** Quality assessment for non-randomized trials

Quality assessment for non-randomized trials	Ren 2017 CCT
A clearly stated aim	2
Inclusion of consecutive patients	2
Prospective data collection	0
Endpoints appropriate to the aim of the study	2
Unbiased assessment of the study endpoint	2
A follow-up period appropriate to the aims of study	2
Less than 5% loss to follow-up	2
Prospective calculation of the sample size	0
An adequate control group	2
Contemporary groups	2
Baseline equivalence of groups	2
Adequate statistical analyses	2
Total score	20

### Outcomes of meta-analysis

It was possible to perform a meta-analysis with nine outcomes (Table 3). There were statistically significant differences between topical TXA and control groups for total blood loss (MD = − 267.53, *P* = 0.00001), drainage volume (MD = − 157, *P* = 0.00001), length of hospital stay (MD = − 1.42, *P* < 0.00001), and postoperative hemoglobin level (MD = 0.95, *P* = 0.0003). There were no statistically significant differences between topical TXA and control groups for blood transfusion rate (RD = − 0.18, *P* = 0.28), wound hematoma (RD = 0.00, *P* = 1.00), wound infection (RD = 0.00, *P* = 1.00), DVT (RD = 0.00, *P* = 1.00), and PE (RD = 0.00, P = 1.00).

**Table 3:** Meta-analysis results

Outcome	Studies	Groups (A/C)	Overall effect			Heterogeneity	
			Effect estimate	95% CI	*p* value	*I*^2^ (%)	*p* value
Total blood loss	2	66/64	− 267.53	− 373.04, − 106.02	0.00001	0	0.38
Drainage volume	4	136/134	− 157.00	− 191.17, − 122.84	0.00001	42	0.16
Blood transfusion rate	4	136/134	− 0.18	− 0.51, 0.15	0.28	94	0.00001
Postoperative hemoglobin level	2	80/80	0.95	0.44, 1.47	0.0003	0	0.43
Hematoma	3	120/120	0.00	− 0.03, 0.03	1.00	0	1.00
Infection	3	120/120	0.00	− 0.03, 0.03	1.00	0	1.00
Deep Vein Thrombosis	2	90/90	0.00	− 0.03, 0.03	1.00	0	1.00
Pulmonary embolism	2	90/90	0.00	− 0.03, 0.03	1.00	0	1.00
Length of hospital stay	2	70/70	− 1.42	− 1.92, − 0.93	0.00001	79	0.03

*A* aminocaproic acid, *C* control, *CI* confidence interval

## Discussion

The intravenous application of TXA has been confirmed as effectively decreasing blood loss and transfusion requirement in spinal surgery [6, 7]. Recently, topical TXA is widely established in hip and knee arthroplasty and successfully reduced postoperative blood loss and blood transfusion requirements [17, 18]. Astedt et al. [19] considered that TXA acts at the active bleeding and clot formation site and not within the circulation itself. But there have been limited studies reporting the efficacy of topical administration in spinal surgery. This is the first meta-analysis to evaluate the efficacy and safety of topical application of TXA in spinal surgery. The most important results of the present meta-analysis were that the topical application of TXA during spinal surgery decreased total blood loss, drainage volume, and length of hospital stay and preserved higher postoperative hemoglobin level. Moreover, no significant difference is noticeable regarding the occurrence of infection, hematoma, DVT, and PE.

Total calculated blood loss ranged from 650 to 2839 ml in adult spine fusion surgery, and transfusion requirements were 50 to 81% without plotting any strategy to reduce hemorrhage [20]. Pooled results indicated that total blood loss and drainage volume in the topical TXA group were significantly lower than that in the control group. Total blood loss included intra-operative blood loss (IBL), post-operative blood loss (PBL), and hidden blood loss (HBL). Xu et al. [15] performed an RCT evaluating the efficacy of topical tranexamic acid in posterior spinal fusion surgeries. They reported that IBL showed no significant difference between two groups. Most of current studies calculated PBL by measuring the amount of “blood” by wound drainage. All included studies reported that topical TXA reduce post-operative drainage volume. These results were consistent with our meta-analysis. The mechanisms of HBL may be hemolysis [21, 22] and loss going into tissue compartments [23]. Smorgick et al. [24] reported that hidden blood loss accounts for 45% of total blood loss. Ren et al. [12] reported that topical TXA effectively reduce HBL following posterior lumbar interbody fusion (PLIF).

The indications for blood transfusion were based on postoperative hemoglobin levels and clinical symptoms of anemia. Although present meta-analysis showed that blood transfusion rate in topical TXA group is lower, there was no significant difference found between the two groups. The reason could be that transfusions trigger varied from different studies. Length of hospital stay is another element in determining the effectiveness of THA and TKA. Recently, two RCTs [11, 15] have reported that topical administration of TXA reduces length of hospital stay in PLIF. This was consistent with our meta-analysis results. The decreased blood loss contributes to not only a lower risk of anemia but also better recovery and shorter hospitalization.

DVT is a common complication following spine surgery, may develop to PE and result in serious complications. In theory, the intravenous application of TXA may enhance the possibility of venous thromboembolism. Present meta-analysis showed that topical application of TXA did not increase the risk of DVT or PE. Bleeding may still occur after wound closed and result in wound hematoma or infection. Topical application of TXA is simple and provides a maximum concentration of TXA at the bleeding field. Present meta-analysis showed that topical TXA did not increase the risk of wound hematoma or infection. Taking these findings together, we conclude that topical TXA is safe in spine surgery.

Several potential limitations should be noted. (1) Only four studies with relatively small sample size were included; (2) subgroup analysis was not performed because of the limited number of included studies, and we could not determine the source of heterogeneity; (3) methodological weaknesses exist in studies; and (4) several outcome parameters were not fully described so that we failed to perform a meta-analysis.

## Conclusion

The present meta-analysis indicated that the topical application of TXA in spinal surgery decreases the total blood loss and drainage volume and preserves higher postoperative hemoglobin level without increasing the risk of infection, hematoma, DVT, and PE. More high-quality randomized controlled trials are required due to the limited quality and data in the evidence currently available.

## Abbreviations

CI: Confidence interval
DVT: Deep vein thrombosis
HBL: Hidden blood loss
IBL: Intra-operative blood loss
MD: Mean difference
MINORS: Methodological index for non-randomized studies
PBL: Post-operative blood loss
PE: Pulmonary embolism
PLIF: Posterior lumbar interbody fusion
RCTs: Randomized controlled trials
RD: Risk difference
TKA: Total knee arthroplasty
TXA: Tranexamic acid
